# Do Rural–Urban Disparities Translate into Nursing Complexity at Hospital Admission? Evidence from a Single Tertiary-Care Pediatric Hospital in Italy

**DOI:** 10.3390/children13060746

**Published:** 2026-05-27

**Authors:** Manuele Cesare, Gianfranco Damiani, Gloria Anderson, Jessica Preziosi, Rosemary Frasso, Leonardo Villani, Nursing and Public Health Group, Vittorio Maio, Antonello Cocchieri

**Affiliations:** 1 A. Gemelli IRCCS University Hospital Foundation, Largo Agostino Gemelli 8, 00168 Rome, Italy; gloria.anderson@guest.policlinicogemelli.it (G.A.); jessica.preziosi@guest.policlinicogemelli.it (J.P.); antonello.cocchieri@policlinicogemelli.it (A.C.); 2 Section of Hygiene, Department of Life Sciences and Public Health, Università Cattolica del Sacro Cuore, Largo Francesco Vito 1, 00168 Rome, Italy; leonardo.villani@unicamillus.org (L.V.); nursingandpublichealthgroup@gmail.com; 3 Hospital Hygiene Unit, A. Gemelli IRCCS University Hospital Foundation, 00168 Rome, Italy; gianfranco.damiani@policlinicogemelli.it; 4 College of Population Health, Thomas Jefferson University, 901 Walnut St., 10th Floor, Philadelphia, PA 19107, USA; rosemary.frasso@jefferson.edu (R.F.); vittorio.maio@jefferson.edu (V.M.); 5 Asano-Gonnella Center for Research in Medical Education and Health Care (CRMEHC), Sidney Kimmel Medical College, Thomas Jefferson University, Philadelphia, PA 19107, USA; 6 Faculty of Medicine and Surgery, UniCamillus-Saint Camillus International University of Health and Medical Sciences, Via di Sant’Alessandro, 00131 Rome, Italy

**Keywords:** pediatric nursing, nursing complexity, health inequities, health status disparities, urbanization, standardized nursing terminology, nursing diagnosis

## Abstract

**Highlights:**

**What are the main findings?**

- In our cohort, pediatric admissions of children from less urbanized areas present a higher clinical burden, reflected in higher DRG weight and a greater number of chronic conditions.
- Despite differences in clinical burden, we found no evidence of an independent association between children’s degree of urbanization and nursing complexity at hospital admission.

**What are the implications of the main findings?**

- Differences in clinical burden associated with children’s geographic origin do not necessarily translate into differences in nursing complexity, highlighting the need to assess these dimensions separately.
- Integrating standardized nursing data into population-level analyses is key to determining whether rural–urban disparities extend to admission-level care needs, ultimately supporting more informed planning of pediatric health services.

**Abstract:**

Background/Objectives: Rural–urban disparities are well documented in pediatric healthcare, influencing access, service organization, and outcomes. However, whether these differences extend to the domain of nursing care remains unclear. This study examined the association between degree of urbanization and nursing complexity among children admitted to a single tertiary-care pediatric hospital, comparing clinical, organizational, and nursing characteristics across geographic groups. Methods: Routinely collected electronic nursing documentation and hospital administrative records were retrospectively examined in a tertiary-care pediatric hospital in Rome, Italy. Children aged 2 to <18 years were consecutively included. Degree of urbanization was defined using the ISTAT-derived DEGURBA classification. The study outcome for nursing complexity corresponded to the number of nursing diagnoses documented at hospital admission. Descriptive analyses and a multivariable negative binomial regression model assessed the association between urbanization and nursing complexity, adjusting for age, sex, DRG weight, number of medical diagnoses, and chronic conditions. Results: Among 1652 pediatric hospitalizations, those from rural areas showed a higher clinical burden, with significantly higher DRG weight and more chronic conditions, but nursing complexity did not differ significantly across geographic groups. In the multivariable model, degree of urbanization was not independently associated with nursing complexity for admissions from towns and suburbs (IRR = 0.93, 95% CI 0.82–1.04, *p* = 0.211) or rural areas (IRR = 1.00, 95% CI 0.84–1.20, *p* = 0.966), compared with those from cities. Higher DRG weight (IRR = 1.11, 95% CI 1.05–1.17) and a greater number of medical diagnoses (IRR = 1.17, 95% CI 1.11–1.25) were positively associated with nursing complexity, whereas the number of chronic conditions showed an inverse association (IRR = 0.90, 95% CI 0.84–0.96). Conclusions: In this single-center sample, rural–urban differences in clinical burden are not accompanied by statistically detectable differences in nursing complexity at hospital admission. Despite a higher clinical burden among children admitted from less urbanized areas, nursing complexity does not appear to differ significantly across geographic groups. Standardized nursing data enhance the ability to compare admission-level care needs across geographic contexts, enabling more precise and stratified population and public health analyses.

## 1. Introduction

Geographic inequalities in healthcare delivery, commonly framed as rural-urban disparities, represent a persistent and well-documented challenge across health systems, influencing access to services, patterns of care, and patient outcomes [1,2,3]. In pediatric populations, rural-urban disparities are particularly relevant, as differences in healthcare availability, socioeconomic conditions, and service organization may influence not only disease trajectories but also the intensity and type of care received [2,4]. Recent evidence indicates that children living in rural or less urbanized areas experience substantial disparities in healthcare access and delivery. They are significantly more likely to receive care in hospitals without dedicated pediatric services and to follow care pathways that differ from those of their urban counterparts. These structural differences have been associated with variations in clinical outcomes and may reflect a higher underlying burden of health needs in rural populations [1,5].

While the influence of geographic context on clinical outcomes and healthcare utilization has been extensively investigated, far less attention has been paid to whether such disparities extend to the domain of nursing care. This represents a critical gap in the literature. Nursing care is a central yet often under-recognized component of healthcare delivery, encompassing continuous patient monitoring, coordination, and response to evolving care needs [6]. However, commonly used indicators of healthcare demand—such as Diagnosis-Related Groups (DRGs), length of stay (LOS), or comorbidity indices—primarily capture medical complexity and resource utilization, offering only limited insight into patient-level nursing care needs [7,8].

In recent years, the increasing availability of standardized nursing data embedded within electronic health records has created new opportunities to objectively quantify nursing care [7,9] and to conduct population-based observational studies [10]. In particular, the number of nursing diagnoses identified early during hospitalization has been used as an operational indicator of nursing complexity in studies relying on standardized nursing data [11]. This measure offers a complementary perspective to traditional clinical indicators, enabling the exploration of dimensions of care that remain largely invisible when relying solely on administrative or medical data [7].

Within this framework, evidence remains limited on whether geographic disparities in pediatric healthcare are reflected in differences in nursing complexity at hospital admission, or whether they are primarily captured by measures of clinical severity and resource utilization. Although prior studies have documented substantial rural-urban inequalities in healthcare access, service organization, and the distribution of nursing resources [12,13], this body of work has largely focused on system-level characteristics rather than individual nursing care needs. As a result, evidence is still limited regarding the extent to which geographic context is associated with variation in nursing complexity at the time of hospital admission.

Accordingly, this study aimed to examine the association between the degree of urbanization of pediatric patients’ area of residence and nursing complexity, measured through standardized nursing diagnoses recorded within the first 24 h of hospitalization. In addition, key clinical and organizational indicators were compared across geographic groups.

## 2. Materials and Methods

### 2.1. Design

The study retrospectively analyzed routinely collected electronic health record (EHR) data from a tertiary-care pediatric hospital located in Rome (Lazio Region), Italy. All eligible hospitalizations occurring between 1 January and 31 December 2022 were included. The reporting of the study was conducted in accordance with the RECORD (REporting of studies Conducted using Observational Routinely-collected health Data) statement [14].

### 2.2. Data Sources

Nursing data were extracted from the Professional Assessment Instrument (PAI), a structured clinical information system integrating standardized nursing documentation into routine clinical practice. At admission, nurses perform an initial patient assessment in accordance with Joint Commission International (JCI) standards [15]. A validated algorithm automatically generates suggested nursing diagnoses [16] based on the Clinical Care Classification (CCC) system [17]. These algorithm-generated nursing diagnoses are intended as candidate outputs to support, rather than replace, nurses’ clinical reasoning. Nurses are required to review the suggested diagnoses and may accept, modify, or reject them when defining the individualized care plan. This approach supports standardized data collection while preserving clinical judgment, enhancing the consistency and comparability of nursing documentation across hospitalizations. Nursing staff at the study hospital receive ongoing training and are subject to quality monitoring related to standardized documentation.

Administrative and clinical variables were collected through the Hospital Discharge Register (Scheda di Dimissione Ospedaliera, SDO) [18], including patients’ area of residence, LOS, DRG weight, and number of medical diagnoses. Nursing and administrative data were integrated at the patient level. Data extraction was performed by the hospital information technology department (ICT), and all records were de-identified prior to analysis.

### 2.3. Participants and Eligibility Criteria

The study sample comprised children and adolescents aged 2 to <18 years consecutively admitted to the study hospital in 2022. For children with more than one admission during the study period, only the first eligible hospitalization was retained and considered as the index hospitalization, in order to avoid multiple observations from the same patient [19]. Admissions with a LOS shorter than 2 days were excluded, since these episodes may reflect short-term pediatric care pathways similar to day-hospital services [7]. Within the organizational model of the study hospital, these admissions follow partially different clinical and nursing care pathways and do not routinely include the full nursing assessment and documentation processes typically performed for ordinary inpatient hospitalizations. Analyses involving degree of urbanization were restricted to patients with a classifiable area of residence within Italy, as the Italian National Institute of Statistics (ISTAT)-derived DEGURBA (Degree of Urbanisation) classification applies only to Italian municipalities (see Section 2.4.1.).

### 2.4. Variables

#### 2.4.1. Exposure

The primary exposure was degree of urbanization of the patient’s area of residence, classified using the standardized Eurostat DEGURBA system [20,21]. DEGURBA is based on population size, density thresholds, and geographic contiguity using a regular grid of 1 km^2^ cells, in accordance with the European Commission methodology (Regulation EU 2017/2391). By relying on grid-based spatial units rather than administrative boundaries, this approach improves cross-national comparability and reduces potential bias related to differences in the size and shape of territorial units [22]. Classification was derived from data provided by the ISTAT [23], which applies the Eurostat DEGURBA methodology using census-based population data. The DEGURBA classification is initially defined at the grid-cell level and subsequently aggregated to the municipality level, with each municipality assigned to a single degree of urbanization category.

Municipalities were categorized as (1) densely populated areas (cities), (2) intermediate-density areas (towns and suburbs), and (3) thinly populated areas (rural areas). According to the DEGURBA methodology, these categories derive from population density and minimum population thresholds applied to clusters of contiguous grid cells: urban centers are defined as clusters with a density ≥ 1500 inhabitants per km^2^ and a total population ≥ 50,000; urban clusters as clusters with a density ≥ 300 inhabitants per km^2^ and a population ≥ 5000; and rural areas as all remaining territories below these thresholds. For municipalities that underwent administrative changes, the degree of urbanization was assigned based on the 2011 census classification to ensure temporal consistency, following ISTAT standard procedures for longitudinal comparability. The exposure was modeled as a categorical variable, with urban areas as the reference group.

#### 2.4.2. Outcome

The outcome measure for nursing complexity corresponded to the number of nursing diagnoses recorded within the first 24 h of hospitalization, reflecting the initial assessment of patient care needs [11].

#### 2.4.3. Covariates

Covariates included age, sex, DRG weight, number of medical diagnoses coded according to the International Classification of Diseases, 9th Revision, Clinical Modification (ICD-9-CM) (the coding system routinely adopted in 2022 within the Italian Hospital Discharge Register for administrative hospital data) [18], and the number of chronic conditions. DRG weight was included as a proxy of hospitalization-level clinical severity and expected resource utilization [7]. Chronic conditions were identified using the Chronic Condition Indicator developed by the U.S. Agency for Healthcare Research and Quality (AHRQ) [24], which classifies each ICD-9-CM diagnosis code as chronic or non-chronic. The number of chronic conditions was quantified as the count of chronic diagnoses per hospitalization. The number of medical diagnoses and the number of chronic conditions were treated as distinct variables to capture overall diagnostic burden and underlying chronic disease burden, respectively. These variables were selected a priori based on established associations with healthcare utilization and nursing complexity [7,25,26,27]. All covariates were measured at the patient level using routinely collected data.

### 2.5. Statistical Analysis

Descriptive statistics summarized demographic, clinical, organizational, and nursing characteristics across DEGURBA categories. Continuous data were described using either mean and standard deviation or median and interquartile range, depending on data distribution, while categorical data were presented as counts and percentages. Between-group differences were assessed using one-way ANOVA for normally distributed continuous variables and Kruskal–Wallis tests for non-normally distributed continuous variables. Categorical variables were compared using chi-square tests or Fisher-Freeman-Halton exact tests, according to expected cell frequencies. Analyses of individual nursing diagnoses were considered exploratory; therefore, *p*-values for these comparisons were interpreted cautiously, and no formal adjustment for multiple comparisons was applied.

The independent association between degree of urbanization and nursing complexity was assessed using a multivariable negative binomial regression, with the number of nursing diagnoses within the first 24 h as the dependent variable. This model was selected due to overdispersion of the count outcome. Models were adjusted for age, sex, DRG weight, number of medical diagnoses, and number of chronic conditions. To assess the robustness of the adjusted estimates under alternative model specifications, additional sensitivity analyses were conducted by re-estimating the multivariable negative binomial regression model using sequential modifications of the covariate structure. Multicollinearity among independent variables was assessed using variance inflation factors (VIFs) and tolerance values, calculated through an auxiliary linear regression model including all covariates. Results were presented as incidence rate ratios (IRRs) with 95% confidence intervals. Statistical significance was set at *p* < 0.05. All analyses were conducted using SPSS version 31.0 for macOS. Geographic visualization was performed using QGIS (version 4.0.0-Norrköping for macOS), based on official Italian administrative boundaries retrieved via the *istat_confini_plugin* (version 1.3.0). Pediatric hospitalizations were aggregated by region of residence and displayed using a choropleth map. Hospitalization counts were classified into quintiles using a quantile (equal count) approach and represented through a sequential color scale.

### 2.6. Ethical Considerations

Ethical approval for this study was granted by the Ethics Committee of the Catholic University of the Sacred Heart (Prot. 0012915/24, ID 6752; 16 May 2024). As this was a retrospective observational study, the dataset was obtained from routinely recorded clinical information available in healthcare records. Parents or legal guardians provided a general consent for the use of anonymized clinical data at admission. In accordance with the Ethics Committee requirements, parents or legal guardians were later contacted to provide specific authorization for the retrospective use of anonymized data. All data were de-identified prior to analysis by the ICT and handled in accordance with the General Data Protection Regulation. The study adhered to the Declaration of Helsinki and Good Clinical Practice.

## 3. Results

### 3.1. Sample Characteristics

Following application of the eligibility criteria, 15 hospitalizations were excluded because the area of residence was outside Italy and could not be classified according to the ISTAT-derived DEGURBA system. The final analytical sample comprised 1652 pediatric hospitalizations.

The mean age at hospitalization was 10.0 years (SD 5.0), and 56.8% (n = 938) were male children. When stratified by degree of urbanization, 50.7% (n = 838) were admissions of children residing in cities, 37.7% (n = 622) in towns and suburbs, and 11.6% (n = 192) in rural areas.

**Figure 1:**
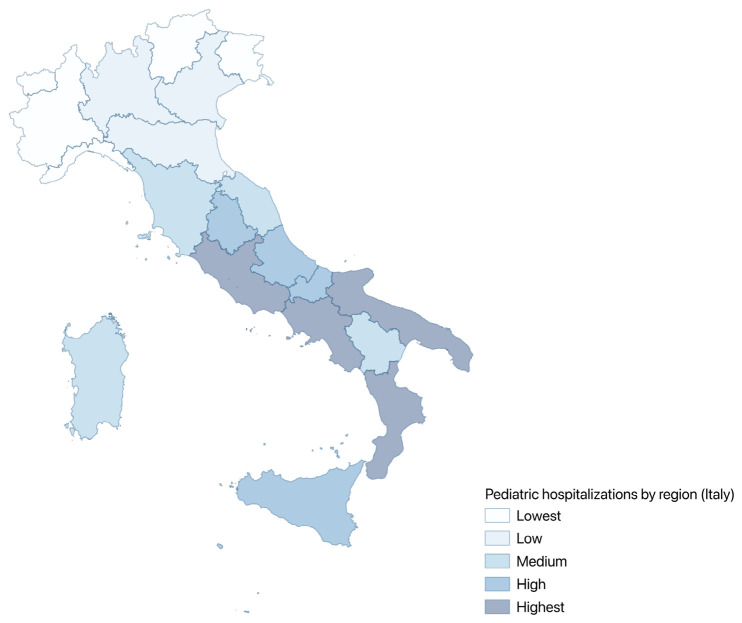
Regions are grouped into quintiles of hospitalization counts, from lowest to highest, as indicated in the legend. Color intensity in-creases with hospitalization frequency. Hospitalization counts by region (ascending order): Piedmont (n = 1), Trentino-Alto Adige/South Tyrol (n = 1), Friuli-Venezia Giulia (n = 1), Veneto (n = 2), Emilia-Romagna (n = 2), Lombardy (n = 6), Marche (n = 10), Tuscany (n = 15), Sardinia (n = 17), Basilicata (n = 21), Umbria (n = 22), Molise (n = 22), Sicily (n = 29), Abruzzo (n = 46), Calabria (n = 76), Campania (n = 81), Apulia (n = 89), Lazio (n = 1211).

Regarding geographic distribution, most records referred to children residing in Central Italy (76.2%), followed by Southern regions (20.3%). Smaller proportions were from the Islands (2.8%), North-West (0.4%), and North-East (0.4%). At the regional level, most records referred to children residing in Lazio (73.3%), consistent with the location of the study hospital, followed by Campania (4.9%), Apulia (5.4%), Calabria (4.6%), and other regions (Figure 1).

The median LOS was 4.0 days (IQR 4) and the median DRG weight was 0.7933 (IQR 0.6316). On average, each hospitalization included 1.9 medical diagnoses (SD 1.2) and 1.1 chronic conditions (SD 1.1). The mean number of nursing diagnoses recorded within the first 24 h of hospitalization was 4.0 (SD 2.7).

### 3.2. Differences in Hospitalization Characteristics by Degree of Urbanization

Differences in demographic, clinical, and organizational characteristics across degree of urbanization categories are presented in Table 1. Demographic characteristics at hospitalization were comparable across groups, with no statistically significant differences in age (*p* = 0.451) or sex distribution (*p* = 0.598).

Among clinical indicators, DRG weight differed significantly across degree of urbanization categories (*p* < 0.001). Admissions from rural areas showed higher DRG weights (median 0.8997, IQR 0.6867) compared with those from cities (median 0.7668, IQR 0.5522) and towns and suburbs (median 0.7933, IQR 0.6779). Similarly, the number of chronic conditions was significantly higher among admissions from rural areas (mean 1.2, SD 1.1) than among those from cities (mean 0.9, SD 1.0; *p* < 0.001).

A trend toward longer LOS was observed among admissions from rural areas (median 5.0 days) compared with other groups (median 4.0 days), although this difference did not reach statistical significance (*p* = 0.063). The number of medical diagnoses and the number of nursing diagnoses documented at admission were comparable across degree-of-urbanization categories (*p* = 0.461 and *p* = 0.114, respectively).

### 3.3. Nursing Complexity by Degree of Urbanization

The distribution of nursing complexity across degree of urbanization categories is shown in Table 2. The mean number of nursing diagnoses within the first 24 h of hospitalization did not differ across groups, with averages of 4.0 (SD 2.7) in cities, 3.8 (SD 2.7) in towns and suburbs, and 4.2 (SD 3.0) in rural areas (*p* = 0.114). Across all geographic groups, the most frequently reported nursing diagnoses were Fall Risk, Infection Risk, Acute Pain, and Sleep Pattern Disturbance, which consistently ranked as the top four diagnoses.

Some differences in the distribution of individual nursing diagnoses were observed across degree-of-urbanization categories. Fall Risk showed a higher proportion in towns and suburbs compared with cities and rural areas (*p* = 0.034). Several diagnoses—including Acute Pain, Sleep Pattern Disturbance, Anxiety, Fluid Volume Deficit, and Body Nutrition Deficit—showed higher proportions in cities compared with towns and suburbs and rural areas (all *p* < 0.05). In contrast, Skin Integrity Alteration and Toileting Deficit showed higher proportions in rural areas compared with cities and towns and suburbs (*p* = 0.034 and *p* = 0.049, respectively). For the remaining nursing diagnoses, distributions were comparable across the three geographic groups.

### 3.4. Multivariable Negative Binomial Regression Analysis of the Association Between Degree of Urbanization and Nursing Complexity

Results from the multivariable negative binomial regression model are presented in Table 3. After adjustment for age, sex, DRG weight, number of medical diagnoses, and number of chronic conditions, no evidence of an independent association between degree of urbanization and nursing complexity emerged in this sample. The estimates did not indicate statistically significant differences for towns and suburbs (IRR = 0.93, 95% CI 0.82–1.04, *p* = 0.211) or rural areas (IRR = 1.00, 95% CI 0.84–1.20, *p* = 0.966), compared with cities.

Among the covariates, higher DRG weight (IRR = 1.11, 95% CI 1.05–1.17, *p* < 0.001) and number of medical diagnoses (IRR = 1.17, 95% CI 1.11–1.25, *p* < 0.001) were independently associated with a higher number of nursing diagnoses at admission. Conversely, the number of chronic conditions showed an inverse association with nursing complexity (IRR = 0.90, 95% CI 0.84–0.96, *p* = 0.001). Age and sex were not significantly associated with the outcome. Collinearity diagnostics did not indicate problematic multicollinearity, with VIFs ranging from 1.01 to 1.63 and tolerance values above 0.60.

### 3.5. Sensitivity Analyses

Sensitivity analyses are reported in Table 4. Re-estimation of the multivariable model after alternatively excluding chronic condition count and DRG weight produced only limited changes in the estimates associated with degree of urbanization. The association between chronic condition count and nursing complexity changed direction after DRG weight was excluded from the model. The estimates associated with degree of urbanization remained stable across alternative model specifications.

## 4. Discussion

This study examined whether rural–urban disparities in pediatric populations translate into differences in nursing complexity at hospital admission. The findings suggest a clear dissociation between clinical burden and early nursing complexity: although children from less urbanized areas presented with a higher clinical burden, degree of urbanization was not independently associated with nursing complexity at admission. This distinction is central to the interpretation of the study, as it indicates that geographic disadvantage may be reflected in the clinical profile of hospitalized children without necessarily corresponding to a higher level of documented nursing complexity during the first 24 h of hospitalization.

These findings are consistent with prior literature showing that rural pediatric populations often experience disadvantages in access to care and service availability, which may contribute to delayed care and, consequently, to a higher baseline clinical burden or severity of illness at hospital admission [1,2,5,28]. In line with this evidence, children from less urbanized areas in our sample showed significantly higher DRG weight and a greater number of chronic conditions, supporting the interpretation that geographic disparities were more strongly expressed in underlying clinical burden than in early nursing care needs. This point is important because it confirms that the rural–urban gradient observed in pediatric health is visible in traditional clinical indicators in our data, but not in the nursing complexity outcome adopted in this study.

The absence of an independent association between degree of urbanization and nursing complexity should therefore not be interpreted as a lack of disparity overall, but rather as evidence that disparities may operate differently depending on the dimension of need being measured. Nursing complexity was measured through nursing diagnoses recorded within the first 24 h, a recognized proxy of nursing care complexity in studies based on standardized nursing data [11]. By definition, this measure captures needs identified during the early phase of hospitalization and is therefore more closely related to immediate and acute care requirements than to the broader longitudinal burden associated with chronic illness or cumulative social disadvantage. Within this perspective, the present findings suggest that early nursing complexity and clinical burden are related but non-equivalent constructs.

This interpretation is further supported by the multivariable model. Higher DRG weight and a greater number of medical diagnoses were positively associated with nursing complexity, indicating that more severe or diagnostically complex hospitalizations tend to generate a greater number of nursing problems identified at admission. These associations support the interpretation that nursing complexity reflects an admission-level dimension of patient need that is connected to, but not fully captured by, traditional clinical indicators.

At the same time, the association between chronic condition count and nursing complexity should be interpreted with particular caution. Sensitivity analyses showed that this association changed from an inverse to a positive association after exclusion of DRG weight from the model, suggesting partial overlap between these variables as indicators of hospitalization-level clinical burden. Therefore, the inverse association observed in the fully adjusted model should be regarded as a model-dependent finding rather than evidence that chronic illness directly reduces nursing complexity. In contrast, the estimates associated with degree of urbanization remained substantially stable across alternative model specifications, supporting the robustness of the main study finding.

Taken together, these findings reinforce the idea that early nursing complexity does not simply mirror cumulative morbidity but instead captures a distinct dimension of patient need. Interpretation of the null association between degree of urbanization and nursing complexity should nevertheless consider the precision of the estimates. In particular, the confidence interval for the rural-versus-city comparison remained compatible with differences ranging from a modest reduction to approximately a 20% increase in the number of nursing diagnoses recorded at admission. Given the observed average of approximately four nursing diagnoses per hospitalization, the upper confidence bound would correspond to less than one additional nursing diagnosis per admission on average. Although such a difference could still be clinically relevant in selected pediatric contexts, especially among high-acuity admissions, the present data did not provide sufficiently precise evidence to confirm a statistically significant association.

Within these precision limits, this distinction is conceptually relevant. One possible expectation was that children from rural or less urbanized areas, because of their higher clinical burden and known barriers in access to services, might also present with higher nursing complexity at admission. However, our results do not support this assumption. Instead, they suggest that nursing complexity cannot be inferred from geographic disadvantage alone. In other words, geographic inequalities appear to shape which children present at admission with a heavier clinical profile, but not necessarily which admissions require more complex nursing assessment and planning in the very early stage of hospitalization. This is arguably the main contribution of the study.

An alternative explanation for this null finding, however, relates to selection bias generated by the single tertiary referral-center setting. The study sample includes only children who reached and were admitted to a tertiary-care pediatric hospital in Rome. Therefore, children from rural areas in this cohort may not represent all rural children with comparable health needs, but rather a selected subgroup whose families were able to access, navigate, or be referred to a highly specialized hospital. Families facing greater socioeconomic deprivation, transportation difficulties, limited access to pediatric specialty services, or weaker referral and care-coordination pathways may have been underrepresented among children who successfully reached the tertiary referral center [29,30,31]. This selection mechanism may have attenuated differences in documented nursing complexity across degree-of-urbanization categories and may partly explain the absence of an independent association between rural residence and nursing complexity. Accordingly, the null result should not be interpreted as definitive evidence that rurality is unrelated to nursing care needs, but rather as a finding observed among children who successfully accessed a tertiary referral center.

Beyond this potential selection mechanism, the pediatric context itself may also contribute to explaining why higher clinical burden in rural children did not translate into higher documented nursing complexity at admission. Children are rarely managed in isolation, and caregivers play a central role in symptom recognition, daily management, and the decision to seek care [32,33,34]. For this reason, the expression of care needs at admission may be influenced not only by the child’s disease burden, but also by the extent to which caregivers have already recognized, interpreted, and managed problems before hospitalization. Because caregiver support is present across geographic contexts, it may partially buffer pre-hospital vulnerability and reduce the immediate visibility of geographic disparities during the initial nursing assessment [35,36]. Specifically, in rural settings, despite greater barriers in access to services [37,38], families may develop adaptive caregiving strategies and rely on strong informal support networks, including friends and community members, to bridge gaps in formal healthcare services [39,40,41,42]. Although these factors were not directly measured in the present study and should therefore be interpreted with caution, they may contribute to explaining why a higher clinical burden in rural children was not paralleled by a higher early documented nursing complexity. These explanations should therefore be interpreted as complementary rather than mutually exclusive.

From a public and population health perspective, these findings highlight a gap in population-level assessment, as traditional indicators fail to fully capture the multidimensional nature of patient needs [7]. More specifically, they show that indicators such as DRG weight, chronic conditions, and medical diagnoses are informative for identifying gradients in clinical burden, but they are insufficient to determine whether such gradients also translate into differences in documented nursing complexity [7]. In this context, standardized nursing data offer a complementary and still underutilized lens to examine how care needs are expressed across populations, with implications for more precise planning and equitable resource allocation. Their added value lies precisely in making it possible to distinguish between differences in illness burden and differences in patient-level nursing complexity, rather than assuming that the two necessarily overlap.

Some limitations should be acknowledged. Because the study was based on data from a single tertiary-care pediatric hospital, the transferability of the findings to different healthcare settings may be limited and may have introduced referral- and access-related selection bias. In particular, children from rural areas included in this cohort may represent a selected subgroup of families able to successfully access a tertiary referral center, potentially underrepresenting the most socioeconomically disadvantaged or geographically isolated rural populations. Furthermore, although the overall sample size was large, the rural subgroup was smaller than the city and town/suburb groups; therefore, modest differences in nursing complexity among rural children may not have been detected with sufficient precision. In addition, nursing complexity was measured through the number of nursing diagnoses recorded within the first 24 h, thus reflecting needs identified at admission rather than the evolution of complexity during hospitalization. Accordingly, the study cannot exclude the possibility that geographic differences may emerge later in the hospital stay, for example, in the intensity, duration, or progression of nursing care needs. Moreover, the use of standardized nursing documentation embedded within electronic health records may have reduced variability in the identification and recording of nursing diagnoses across patient groups. Consequently, the absence of statistically significant differences in nursing complexity should not be interpreted as definitive evidence of clinical equivalence between geographic groups, as part of the null finding may reflect attenuation of variability related to the algorithm-supported documentation process. In addition, although nurses could critically review, modify, or reject algorithm-generated candidate nursing diagnoses, the retrospective design did not allow us to assess how consistently this clinical scrutiny was applied in practice. Therefore, we cannot exclude the possibility that some suggested diagnoses were accepted with limited critical reassessment, which may have influenced the number and distribution of documented nursing diagnoses and may therefore represent a potential threat to the internal validity of the outcome measurement. Finally, DEGURBA provides a standardized measure of urbanization, but it does not account for other relevant contextual dimensions, such as socioeconomic deprivation, local service availability, or caregiver capacity, which may partly shape the relationship between geography, illness burden, and care needs [30,43,44,45]. This is particularly relevant in pediatric populations, where the pathway from geographic context to hospitalization is mediated by families, service accessibility, and local organizational factors that are not fully captured by municipality-level classification alone. In the Italian context, this limitation is further underscored by evidence of substantial pediatric interregional healthcare mobility, particularly from less resourced areas toward better equipped regions, suggesting that the place of hospitalization may not fully correspond to the patient’s area of residence [46].

Despite these limitations, this study offers a novel contribution by applying standardized nursing data to the study of geographic inequalities in pediatric populations. Its added value lies in supporting the interpretation that nursing complexity may capture a dimension of patient need that is not fully reflected by geographic or clinical indicators alone. This distinction is both conceptually and operationally relevant, as it supports the use of standardized nursing data to better characterize and compare patient-level care needs across geographic contexts.

Future studies should therefore consider stratifying analyses by patterns of healthcare mobility or regional service availability and should specifically investigate how referral, transportation, socioeconomic, and access-related selection mechanisms may influence which rural children successfully reach tertiary-care pediatric hospitals to better disentangle how geographic context and access to care influence clinical burden and the early expression of nursing complexity at hospital admission. They should also extend the use of standardized nursing complexity measures across care settings to examine how nursing complexity evolves during transitions between hospital and community care, and whether geographic disparities become more evident beyond the point of admission. In addition, future research should incorporate caregiver and social-context variables, examine the longitudinal evolution of nursing complexity during hospitalization, and test whether similar patterns are observed across different healthcare settings and populations.

## 5. Conclusions

In this single-center tertiary-care referral cohort, differences according to degree of urbanization are reflected in clinical burden but not in statistically detectable differences in nursing complexity at hospital admission. Despite higher DRG weight and more chronic conditions among children admitted from less urbanized areas, early documented nursing complexity does not appear to differ significantly across geographic groups. Standardized nursing data may help distinguish illness burden from hospitalization-level nursing care needs, ultimately supporting more precise and informed pediatric health services research and planning.

## Figures and Tables

**Table 1:** Differences in hospitalization characteristics by degree of urbanization.

Variable	Total Sample (N = 1652)	Hospitalizations Among Children Residing in Cities (n = 838)	Hospitalizations Among Children Residing in Towns and Suburbs (n = 622)	Hospitalizations Among Children Residing in Rural Areas (n = 192)	*p*-Value ^a^
Age (mean, SD)	10 (5.0)	10.2 (4.9)	9.9 (4.9)	9.8 (5.0)	0.451
Male (n; %)	938 (56.8)	466 (55.6)	359 (57.7)	113 (58.9)	0.598
LOS (median, IQR)	4.0 (4)	4.0 (4)	4.0 (4)	5.0 (4.0)	0.063
DRG weight (median, IQR)	0.7933 (0.6316)	0.7668 (0.5522)	0.7933 (0.6779)	0.8997 (0.6867)	<0.001
Number of medical diagnoses (mean, SD) (ICD-9-CM)	1.9 (1.2)	1.8 (1.1)	1.9 (1.2)	1.9 (1.2)	0.461
Number of chronic conditions (mean, SD)	1.1 (1.1)	0.9 (1.0)	1.1 (1.0)	1.2 (1.1)	<0.001
Number of NDs (mean, SD)	4.0 (2.7)	4.0 (2.7)	3.8 (2.7)	4.2 (3.0)	0.114

^a^ One-way ANOVA for age, number of medical diagnoses, number of chronic conditions, and number of NDs; Kruskal–Wallis test for LOS and DRG weight; chi-square test for sex distribution across degree-of-urbanization categories. Legend: SD, standard deviation; LOS, length of stay; IQR, interquartile range; DRG, diagnosis-related group; ICD-9-CM, International Classification of Diseases, 9th Revision, Clinical Modification; NDs, nursing diagnoses.

**Table 2:** Distribution of nursing diagnoses recorded at admission by degree of urbanization.

Variable	Total Diagnoses (N = 6539) n (%)	Rank	Hospitalizations Among Children Residing in Cities (n = 3383) n (%)	Rank	Hospitalizations Among Children Residing in Towns and Suburbs (n = 2355) n (%)	Rank	Hospitalizations Among Children Residing in Rural Areas (n = 801) n (%)	Rank	*p*-Value ^a^
Fall Risk	1316 (20.1)	1	647 (19.1)	1	508 (21.6)	1	161 (20.1)	1	0.034
Infection Risk	1107 (16.9)	2	562 (16.6)	2	410 (17.4)	2	135 (16.9)	2	0.526
Acute Pain	805 (12.3)	3	433 (12.8)	3	273 (11.6)	3	99 (12.4)	3	0.009
Sleep Pattern Disturbance	576 (8.8)	4	315 (9.3)	4	188 (8.0)	4	73 (9.1)	4	0.009
Injury Risk	340 (5.2)	5	172 (5.1)	6	124 (5.3)	5	44 (5.5)	5	0.670
Anxiety	304 (4.6)	6	176 (5.2)	5	104 (4.4)	7	24 (3.0)	10	0.009
Skin Integrity Impairment Risk	304 (4.6)	6	140 (4.1)	8	123 (5.2)	6	41 (5.1)	6	0.174
Fluid Volume Deficit	250 (3.8)	7	143 (4.2)	7	77 (3.3)	9	30 (3.7)	8	0.046
Feeding Deficit	248 (3.8)	8	128 (3.8)	9	88 (3.7)	8	32 (4.0)	7	0.663
Physical Mobility Impairment	188 (2.9)	9	100 (3.0)	10	63 (2.7)	10	25 (3.1)	9	0.420
Urinary Elimination Alteration	156 (2.4)	10	84 (2.5)		52 (2.2)		20 (2.5)		0.497
Breathing Pattern Impairment	152 (2.3)		77 (2.3)		57 (2.4)		18 (2.2)		0.996
Body Nutrition Deficit	106 (1.6)		66 (2.0)		26 (1.1)		14 (1.7)		0.015
Aspiration Risk	89 (1.4)		38 (1.1)		40 (1.7)		11 (1.4)		0.277
Skin Integrity Alteration	82 (1.3)		43 (1.3)		23 (1.0)		16 (2.0)		0.034
Bathing/Hygiene Deficit	71 (1.1)		39 (1.2)		23 (1.0)		9 (1.1)		0.646
Activity intolerance	61 (0.9)		35 (1.0)		19 (0.8)		7 (0.9)		0.531
Constipation	61 (0.9)		27 (0.8)		26 (1.1)		8 (1.0)		0.589
Dressing/Grooming Deficit	54 (0.8)		28 (0.8)		21 (0.9)		5 (0.6)		0.859
Swallowing Impairment	46 (0.7)		23 (0.7)		18 (0.8)		5 (0.6)		0.973
Airway Clearance Impairment	41 (0.6)		20 (0.6)		16 (0.7)		5 (0.6)		0.968
Noncompliance	30 (0.5)		10 (0.3)		16 (0.7)		4 (0.5)		0.127
Activity Intolerance Risk	21 (0.3)		11 (0.3)		9 (0.4)		1 (0.1)		0.739
Body Nutrition Excess	20 (0.3)		8 (0.2)		9 (0.4)		3 (0.4)		0.584
Diarrhea	16 (0.2)		7 (0.2)		9 (0.4)		–		0.172
Individual Coping Impairment	14 (0.2)		9 (0.3)		4 (0.2)		1 (0.1)		0.637
Tissue Perfusion Alteration	13 (0.2)		7 (0.2)		5 (0.2)		1 (0.1)		1.000
Bowel Incontinence	12 (0.2)		7 (0.2)		4 (0.2)		1 (0.1)		0.918
Fatigue	11 (0.2)		4 (0.1)		4 (0.2)		3 (0.4)		0.245
Toileting Deficit	11 (0.2)		5 (0.1)		2 (0.1)		4 (0.5)		0.049
Body Image Disturbance	9 (0.1)		5 (0.1)		4 (0.2)		–		0.802
Social Interaction Alteration	8 (0.1)		5 (0.1)		2 (0.1)		1 (0.1)		0.785
Fear	7 (0.1)		5 (0.1)		2 (0.1)		–		0.651
Chronic Pain	6 (0.1)		2 (0.1)		4 (0.2)		–		0.415
Confusion	4 (0.1)		2 (0.1)		2 (0.1)		–		1.000

^a^ chi-square test or Fisher-Freeman-Halton exact test, according to expected cell frequencies.

**Table 3:** Multivariable negative binomial regression analysis of the association between degree of urbanization and nursing complexity.

Variable	IRR	95% CI	*p*-Value
Towns and suburbs vs. cities	0.93	0.82–1.04	0.211
Rural areas vs. cities	1.00	0.84–1.20	0.966
Age	1.00	0.99–1.02	0.494
Male sex	1.04	0.93–1.16	0.471
DRG weight	1.11	1.05–1.17	<0.001
Number of medical diagnoses (ICD-9-CM)	1.17	1.11–1.25	<0.001
Number of chronic conditions	0.90	0.84–0.96	0.001

Reference categories: cities for degree of urbanization and female sex. Legend: IRR, incidence rate ratio; CI, confidence interval; DRG, diagnosis-related group; ICD-9-CM, International Classification of Diseases, 9th Revision, Clinical Modification.

**Table 4:** Sensitivity analyses of the multivariable negative binomial regression model.

Variable	Fully Adjusted Model IRR (95% CI)	*p*-Value	Model Excluding Chronic Condition Count IRR (95% CI)	*p*-Value	Model Excluding DRG Weight IRR (95% CI)	*p*-Value
Towns and suburbs vs. cities	0.93 (0.82–1.04)	0.211	0.93 (0.78–1.12)	0.442	0.91 (0.76–1.09)	0.304
Rural areas vs. cities	1.00 (0.84–1.20)	0.966	1.02 (0.86–1.22)	0.802	0.99 (0.83–1.18)	0.876
Age	1.00 (0.99–1.02)	0.494	1.01 (0.99–1.02)	0.386	1.01 (0.99–1.02)	0.260
Male sex	1.04 (0.93–1.16)	0.471	0.97 (0.87–1.08)	0.576	0.96 (0.86–1.07)	0.478
DRG weight	1.11 (1.05–1.17)	<0.001	1.12 (1.06–1.18)	<0.001	—	—
Number of medical diagnoses	1.17 (1.11–1.25)	<0.001	1.11 (1.06–1.16)	<0.001	1.14 (1.09–1.19)	<0.001
Number of chronic conditions	0.90 (0.84–0.96)	0.001	—	—	1.14 (1.09–1.19)	<0.001

Legend: IRR, incidence rate ratio; CI, confidence interval; DRG, diagnosis-related group.

## Data Availability

The data presented in this study are available on request from the corresponding author due to privacy, legal, and ethical reasons.

## Abbreviations

The following abbreviations are used in this manuscript:

AHRQ	Agency for Healthcare Research and Quality
ANOVA	Analysis of Variance
CCC	Clinical Care Classification
CI	Confidence Interval
DEGURBA	Degree of Urbanisation
DRG	Diagnosis-Related Group
EHR	Electronic Health Record
EU	European Union
ICD-9-CM	International Classification of Diseases, 9th Revision, Clinical Modification
ICT	Information and Communication Technology
IQR	Interquartile Range
IRR	Incidence Rate Ratio
ISTAT	Italian National Institute of Statistics
JCI	Joint Commission International
LOS	Length of Stay
ND	Nursing Diagnosis
PAI	Professional Assessment Instrument
QGIS	Quantum Geographic Information System
RECORD	REporting of studies Conducted using Observational Routinely-collected health Data
SD	Standard Deviation
SDO	Scheda di Dimissione Ospedaliera
SPSS	Statistical Package for the Social Sciences
U.S.	United States
VIF	Variance Inflation Factor
